# Testimony in Favor of Dr. Arthur's Method

**Published:** 1873-06

**Authors:** Theodore F. Chupein


### ARTICLE IX.

### *Testimony in favor of Dr Arthur's Method.*

BY THEODORE F. CHUPEIN, D D. S.

During the late war between the States I was sent for by the General commanding the troops, where my company was stationed and doing garrison duty, to render him the service of filling a tooth. I had never thought of taking my dental instruments into camp, but after this request from the General, (which gave me a short furlough, a respite all soldiers were glad to obtain,) to have me operate for him, I kept my instrument case in camp, and operated to a considerable extent for the officers and men in the various companies and regiments stationed around.

The question of filling teeth with gold foil was one of no little pecuniary outlay, even in "Confederate money," for at the time at which I write I paid "*twelve hundred dollars* for *an ounce of gold foil*," bought at Atlanta, Georgia. I was therefore constrained to use tin foil to a considerable extent, and even when this got low and was giving out, to use the chisel and the file, the latter being very often so dull from continual use, notwithstanding frequent sharpenings with the aid of sulphuric acid, that considerable time and torture was used to effect the operation. All cases therefore, of superficial decay, wherever situated, were consigned to the tender mercies of the file and chisel; for men were ever then on the move, and "*we could not wait for cavities to get larger.*" Being in an artillery company I had the advantage of the portable forge, wherein I could renew my tools. This, at least, kept my chisels in order, it lent me no aid with my files. I have within the last month seen cases I had treated *nine years ago*, of superficial caries removed on the buccal and masticating surfaces of the molars with the chisel, and on the proximate surfaces of other teeth with the file, in the most perfect state of preservation, looking as white and as beautiful as if they had never been touched by decay.

I had always been taught in operating on the front upper teeth first to seperate them, and, when well separated, to start the file well under, so as to remove a good portion of the palatine and proximate surface, thus securing a self-cleansing space when the teeth came together again. All the teeth that I treated in this way, in the army, which have come under my observation, exhibit a perfect freedom from decay. I do not know if I can call this "testimony in favor of Dr. Arthur's method," for as *I* understand, his consists of the use of the chisel alone on the proximate surfaces of all the teeth, so as to secure self-cleansing space; but if the operation be performed with a chisel or file, and the result be the same, the testimony as to the efficiency of such separations holds good.—*Dental Times.*

